# Expression Profiling of Differentiating Emerin-Null Myogenic Progenitor Identifies Molecular Pathways Implicated in Their Impaired Differentiation

**DOI:** 10.3390/cells6040038

**Published:** 2017-10-22

**Authors:** Ashvin Iyer, Adam J. Koch, James M. Holaska

**Affiliations:** 1 Department of Biomedical Sciences, Rm 534, Cooper Medical School of Rowan University, 401 South Broadway St., Camden, NJ 08028, USA; aiyer@mail.usciences.edu; 2 Department of Pharmaceutical Sciences, University of the Sciences, Philadelphia, PA 19104, USA; 3 Committee on Genetics, Genomics and Systems Biology, University of Chicago, Chicago, IL 60637, USA; ajkoch@ucsd.edu

**Keywords:** emerin, Emery-Dreifuss muscular dystrophy, myogenic differentiation, lamin

## Abstract

Mutations in the gene encoding emerin cause Emery-Dreifuss muscular dystrophy (EDMD), a disorder causing progressive skeletal muscle wasting, irregular heart rhythms and contractures of major tendons. RNA sequencing was performed on differentiating wildtype and emerin-null myogenic progenitors to identify molecular pathways implicated in EDMD, 340 genes were uniquely differentially expressed during the transition from day 0 to day 1 in wildtype cells. 1605 genes were uniquely expressed in emerin-null cells; 1706 genes were shared among both wildtype and emerin-null cells. One thousand and forty-seven transcripts showed differential expression during the transition from day 1 to day 2. Four hundred and thirty-one transcripts showed altered expression in both wildtype and emerin-null cells. Two hundred and ninety-five transcripts were differentially expressed only in emerin-null cells and 321 transcripts were differentially expressed only in wildtype cells. DAVID, STRING and Ingenuity Pathway Analysis identified pathways implicated in impaired emerin-null differentiation, including cell signaling, cell cycle checkpoints, integrin signaling, YAP/TAZ signaling, stem cell differentiation, and multiple muscle development and myogenic differentiation pathways. Functional enrichment analysis showed biological functions associated with the growth of muscle tissue and myogenesis of skeletal muscle were inhibited. The large number of differentially expressed transcripts upon differentiation induction suggests emerin functions during transcriptional reprograming of progenitors to committed myoblasts.

## 1. Introduction

Mutations in the gene encoding emerin cause X-linked Emery-Dreifuss muscular dystrophy (EDMD), an inherited disorder causing progressive skeletal muscle wasting, irregular heart rhythms, and contractures of major tendons [1,2,3,4]. The skeletal muscle wasting seen in EDMD is predicted to be caused by impaired differentiation of skeletal muscle stem cells and inefficient skeletal muscle regeneration. Skeletal muscle necrosis is rarely seen in EDMD patients; increased skeletal muscle fiber permeability is also rarely seen [5]. Severely affected EDMD patients show extensive fibrosis caused by the inability to properly regenerate and repair damaged skeletal muscle. Supporting this hypothesis, emerin-null mice exhibit delayed skeletal muscle regeneration and repair, motor coordination defects, and mild atrioventricular conduction defects [6,7]. Skeletal muscle biopsies from EDMD patients and emerin-null mice showed compensatory upregulation of skeletal muscle regeneration genes [6,8]. Further, emerin-null myogenic progenitors and emerin-downregulated C2C12 myoblasts exhibit impaired differentiation and myotube formation [9,10,11]. The coordinated temporal expression of important differentiation genes, including *MyoD*, *Myf5*, *Pax3* and *Pax7*, is disrupted in emerin-null myogenic progenitors [12] due to the inability of the genome to properly reorganize during differentiation [6,8,13]. These genomic organizational changes in emerin-null cells combined with the failure of the genome to properly reorganize during emerin-null myogenic differentiation supports the hypothesis that emerin-null myogenic progenitors fail to reprogram their transcriptome upon differentiation cues.

Emerin is a ubiquitously expressed integral inner nuclear membrane protein [14,15,16] with reported roles in nuclear structure, chromatin architecture, genomic organization, cell signaling, and gene expression [11,12,13,17,18,19,20,21,22,23,24]. Growing evidence supports a role for emerin in regulating the expression or activity of key components of signaling pathways important for myogenic differentiation. Wnt, IGF-1, TGF-β, and Notch signaling pathways are important for myogenic differentiation and muscle regeneration pathways, were disrupted in emerin-null myogenic progenitors [13,25,26,27,28,29,30,31]. The Wnt, IGF-1, TGF-β, and Notch pathways have well-established roles in maintaining satellite cell quiescence, satellite cell activation and myogenic differentiation after injury [32,33,34,35].

The JNK, p38 MAPK, ERK and NF-κB signaling pathways were also disrupted in emerin-downregulated myoblasts [36,37] and in emerin-null myogenic progenitors [13]. Proper temporal regulation of p38 MAPK signaling is necessary for myogenic differentiation [38,39,40]. The p38 MAPK pathway was activated in emerin-null myogenic progenitors [13]. The ERK pathway was upregulated in emerin-null cells, emerin-null myogenic progenitors and lamin-A R453W mutant cells, an EDMD2-causing lamin A mutation [13,36,37,41]. ERK inhibition rescued the differentiation of mouse emerin-null myogenic progenitor cells [11] and emerin-downregulated C2C12 myoblasts [10].

These previous studies support a model whereby disruption of myogenic signaling pathways in emerin-null and emerin or lamin mutant myoblasts is responsible for their impaired differentiation. Here we performed RNA sequencing at key transitional points during differentiation of wildtype or emerin-null myogenic progenitors to identify transcriptome-wide changes in gene expression. Bioinformatic approaches were then used to identify molecular pathways and networks implicated in the impaired differentiation of emerin-null myogenic progenitors. Interestingly, we identify the transition from cell cycle exit as a key transcriptional reprogramming step during myogenic differentiation that fails to occur in emerin-null myogenic progenitors.

## 2. Materials and Methods

### 2.1. Cell Culture

Wildtype and emerin-null H2K mouse myogenic progenitors were a generous gift from Tatiana Cohen and Terry Partridge (Children’s National Medical Center, Washington, DC, USA) and were isolated as previously described [13]. Proliferating H2Ks were grown and differentiated as previously described [12]. Proliferating myogenic progenitors were grown in proliferative media consisting of complete high-glucose DMEM (Invitrogen, Carlsbad, CA, USA) supplemented with 20% heat-inactivated FBS (Invitrogen), 2% chick embryo extract (Accurate Chemical), 2% l-glutamine (Invitrogen), 1% penicillin–streptomycin (Invitrogen) and 20 units/mL γ-interferon (Millipore, Burlington, MA). Proliferating cells were plated on gelatin at a density of approximately 650 cells/cm^2^ and grown at 33 °C and 10% CO_2_. Differentiating cells were plated on gelatin at a density of 25,000 cells/cm^2^ in proliferative conditions for 24 h (Day 0), then switched to differentiation media consisting of DMEM supplemented with 5% horse serum (Invitrogen) and 2% l-glutamine, and grown at 37 °C and 5% CO_2_. Cells between passages 5 and 8 were used for all analyses.

### 2.2. RNA-seq

Total RNA was isolated from 2 million wildtype or emerin-null H2Ks at each day of differentiation using the miRNeasy Mini Kit (Qiagen, Germantown, MD, USA, product #217004) and processed according to manufacturer’s protocol. RNA was isolated from three independent cell culture plates for each sample. The University of Chicago Functional Genomics Facility performed library construction and sequencing using the Illumina HiScan-SQ. Quality control of raw sequence was performed with FastQC and reads were mapped to the mouse genome (mm10) with Tophat following established protocols [42]. Differential expression analysis was done using Cuffdiff according to established protocols [43]. Transcripts were considered to be significantly differentially expressed if the *q*-value <0.05. The *q*-value is a *p*-value corrected for the False Discovery Rate (FDR) and represents the standard in the field for determining significance. All differentially expressed transcripts were also >1.5-fold increased or decreased.

### 2.3. Pathway Analysis

Selected gene lists (e.g., day 0 vs. day 1) were submitted to the STRING protein interaction database (http://string-db.org/) or the DAVID gene ontology database (http://david.abcc.ncifcrf.gov/). For Ingenuity Pathway Analysis differentially expressed transcripts obtained from RNA sequencing Analysis of proliferating and differentiating (0 h, 24 h and 48 h) wildtype and emerin-null cells were functionally analyzed based on the Ingenuity pathway analysis (IPA) software. This software is based on computer algorithms that analyze the functional connectivity of the genes from information obtained within the IPA database. The “core analysis” function feature in the software was used to interpret the differentially expressed data, which included biological processes, canonical pathways, upstream transcriptional regulators, and gene networks. Gene lists containing differentially expressed genes in the form of log2 expression values from each observation time point for both cell lines was uploaded and analyzed individually. Each eligible gene identifier was then mapped to its corresponding gene object in the Ingenuity Pathway Knowledge Base. The default background settings with no data cut-off filter in the log2 expression values was used for all analysis. Causal network analysis, which is a part of the Advanced Analytics package of IPA, was not utilized for our analysis. Biological Functions and canonical pathways with *p*-value < 0.05 (Fischer’s exact test) were considered to be statistically significant. This calculates the probability that the assigned biological functions and pathways were not due to random chance. The activation *Z*-score was calculated to predict activation or inhibition of transcriptional regulators based on published findings accessible through the Ingenuity knowledge base. Regulators with *Z*-score greater than 2 or less than −2 were considered to be significantly activated or inhibited.

### 2.4. EdU Incorporation and Immunofluorescence Microscopy

Wildtype and emerin-null cells were grown on glass coverslips coated with gelatin and collected at each day of myogenic differentiation. Proliferating or differentiating myogenic progenitors were treated with 10 µM EdU in DMSO and incubated for two hours. The cells were then fixed with 3.7% formaldehyde in PBS for 15 min, washed three times with PBS, and stored at 4 °C with 0.1% sodium azide in PBS until cells were processed per manufacturer instructions (ThermoFisher Scientific, Waltham, MA, USA). The cells were permeabilized in 0.5% Triton in PBS for 20 min, washed 3 times with 3% BSA in PBS and treated with a Click-IT EdU reaction cocktail. Cells were blocked for one hour at room temperature with 3% BSA in PBS containing 0.1% Triton. Nuclei were stained with DAPI and the cells were stored in PBS with 0.1% sodium azide.

Cells were viewed on a Zeiss Axioskop microscope and images were acquired using a QImaging Retiga EXI camera controlled by iVision (BioVision Technologies, Exton, PA, USA) software running on an iMac. Multiple sections of each well were used to obtain images. Each field had approximately 100–200 cells per field and a total of 1000–2000 nuclei were analyzed for each experiment. Nuclei and cells were counted using ImageJ. There were three wells for each treatment in a given experiment for each biological replicate; at least three biological replicates were performed for each treatment. The total number of EdU-positive cells was divided by the total number of nuclei in an image to yield the percentage of cells in S-phase to determine cell cycle exit.

### 2.5. qPCR

Total RNA was prepared as above and cDNA was generated from RNA using MMLV Reverse Transcriptase (Invitrogen, product # 28025013) per manufacturer instructions for individual qPCRs. qPCR was performed using a combination of individual primer sets (see Table 1 for primer sequences) and SYBR GreenER qPCR SuperMix (Invitrogen, product #11761-500). Relative expression was determined by comparing each experimental to the housekeeping genes GAPDH or Oaz1, as previously described [13].

### 2.6. Data Sharing Statement

RNA sequencing data is available through the NCBI Gene Expression Omnibus (Accession number GSE104560).

## 3. Results

### 3.1. Emerin-Null Myogenic Progenitor Cells Display Impaired Myogenic Differentiation

**Figure 1:**
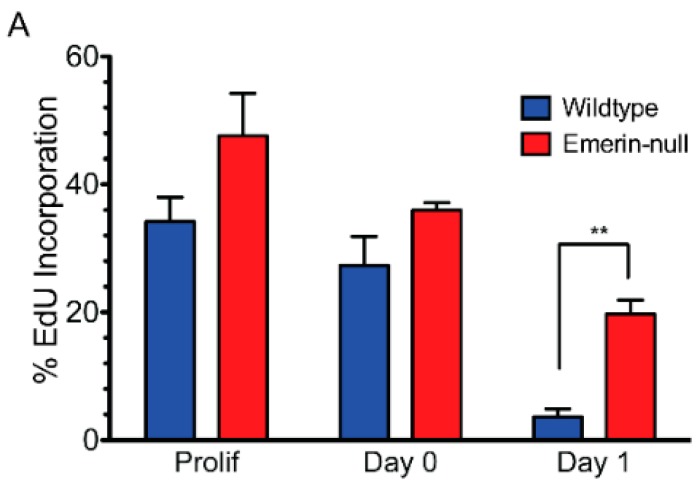

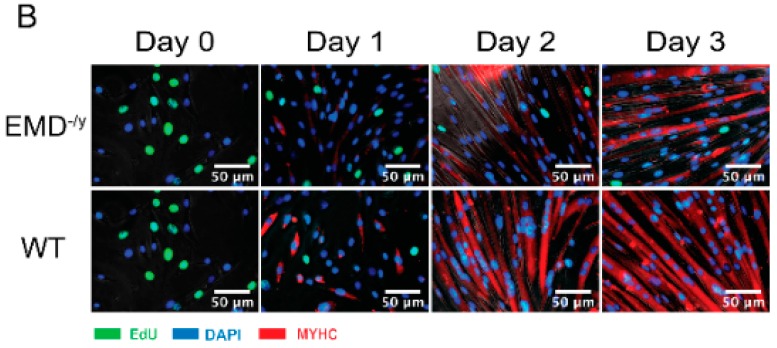
H2K myogenic progenitors have impaired differentiation and delayed cell cycle exit. (**A**) Wildtype (blue) or emerin-null (red) myogenic progenitors were induced to differentiate by serum withdrawal and differentiation was assessed every 24 h. Cell cycle withdrawal was monitored by measuring the incorporation of EdU. ** *p* < 0.01. (**B**) Representative images of wildtype (WT) and emerin-null myogenic progenitors (EMD^−/y^) during differentiation. EdU is shown in green, nuclei are blue and myosin heavy chain (MyHC) is shown in red.

Emerin-null myogenic progenitors were plated at high-density and differentiation was induced by serum withdrawal. Three assays were used to monitor myogenic differentiation: cell cycle exit, myosin heavy chain (MyHC) expression, and cell fusion into myotubes. Incorporation of EdU into DNA of proliferating cells was used to measure the percent of cells in the cell cycle. Emerin-null myogenic progenitor cells had delayed cell cycle withdrawal, as, after 24 h, 19.7% of myogenic progenitors were still cycling compared to 3.6% of wildtype progenitors (*p* = 0.003; Figure 1A,B), consistent with previous studies [11]. These previous studies also showed that expression of myosin heavy chain and myotube formation was significantly impaired.

### 3.2. Emerin-Null Myogenic Progenitor Cells Show Extensive Transcriptional Changes Compared to Wildtype Cells at Each Day of Myogenic Differentiation

To identify putative genes or molecular pathways responsible for the impaired differentiation of emerin-null myogenic progenitors, high-throughput RNA sequencing (RNAseq) was done on differentiating wildtype and emerin-null H2K myogenic progenitors. RNAseq was done on proliferating myogenic progenitors and every day during differentiation for four days, at which time wildtype progenitors formed mature myotubes. These time points were chosen because they represent key transitions during myogenic differentiation: cell cycle withdrawal (day 0), differentiation commitment (day 1), myocyte fusion (days 2–3), myotube maturation (day 3) and mature myotube formation (day 4). RNA was isolated from 2 million wildtype or emerin-null cells for each time point and three biological replicates were done for each time point. RNA was sequenced on the Illumina HiScan-SQ. Sequencing data is available through the NCBI Gene Expression Omnibus (Accession number GSE104560).

**Figure 2:**
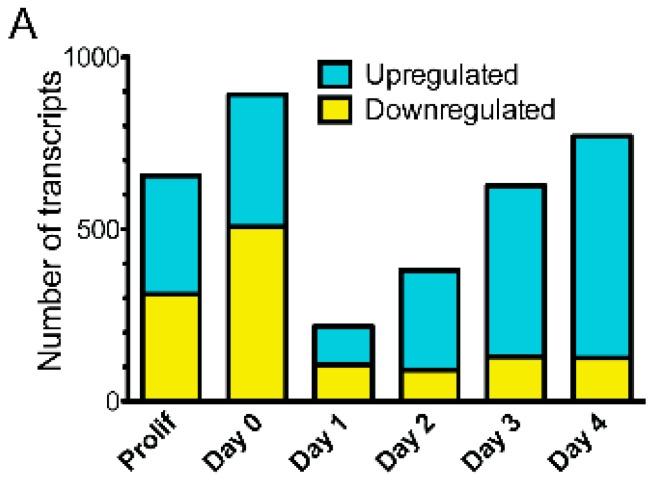

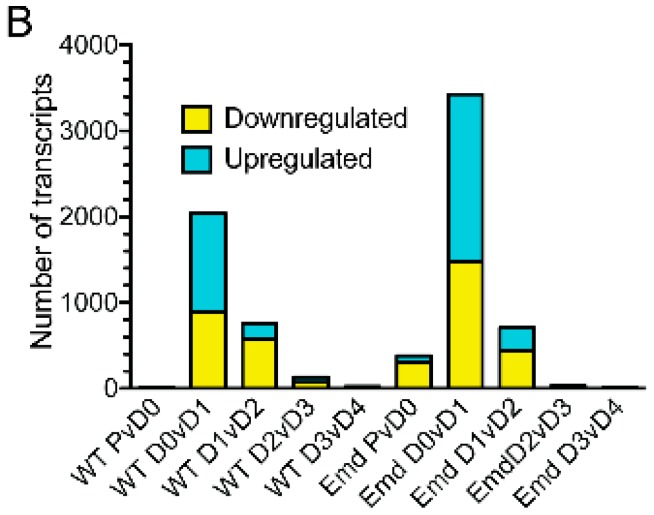
The coordinated temporal expression of thousands of transcripts was altered at each ‘step’ during differentiation of emerin-null myogenic progenitors. (**A**) The number of differentially expressed transcripts between wildtype and emerin-null progenitors at each day during differentiation are shown. (**B**) Comparison of differentially expressed transcripts between consecutive days of differentiation for wildtype (WT) or emerin-null (Emd) myogenic progenitors. P, proliferating cells, D0, day 0; D1, day 1; D2, day 2; D3, day 3; D4, day 4.

Each day of emerin-null myogenic progenitor differentiation was compared to wildtype differentiation to obtain gene expression changes in emerin-null progenitors at each static differentiation step. Transcripts were considered to be significantly differentially expressed if the *q*-value <0.05. 656 differentially expressed (DE) transcripts were seen in proliferating emerin-null myogenic progenitors. During differentiation of emerin-null progenitors, 891 transcripts were differentially expressed at day 0, 219 transcripts were differentially expressed at day 1, 381 transcripts were differentially expressed at day 2, 627 transcripts were differentially expressed at day 3 and 770 transcripts were differentially expressed at day 4 (Figure 2A). The average number of transcripts identified in each comparison was 29,377 ± 423.

### 3.3. Emerin-Null and Wildtype Myogenic Progenitors Diverge Extensively in Transcript Expression During the Transitions from Days 0–2 of Myogenic Differentiation

To better understand how the gene expression program changed at each major transition during differentiation, the transcriptome of wildtype or emerin-null myogenic progenitors from each day of differentiation was compared to the previous day. Transcripts were considered to be significantly differentially expressed if the *q*-value <0.05. It was expected that the expression of a large number of genes would change during differentiation and that failure in the activation or repression of selected transcripts would occur in emerin-null progenitors to cause impaired differentiation. Very few changes in expression were seen upon plating wildtype progenitors at high-density for differentiation, as only sixteen transcripts were differentially expressed (Day 0, Figure 2B). Cell cycle withdrawal initiated the greatest gene expression changes, as 2046 transcripts were differentially expressed between day 0 and day 1 during wildtype progenitor differentiation. The transition to committed myocytes caused 752 genes to be differentially expressed. Myotube formation and maturation resulted in 133 and 24 transcripts to be differentially expressed, respectively. Thus, the biggest transcriptional changes in wildtype myogenic progenitor differentiation occur upon differentiation induction and cell cycle withdrawal. There were an average of 29,261 ± 368 transcripts identified for each comparison.

Differentiating emerin-null myogenic progenitors had a greater number of differentially expressed transcripts at all transitions. 382 transcripts were differentially expressed upon plating proliferating emerin-null myogenic progenitors at high-density (Figure 2B; day 0). 3,426 transcripts were differentially expressed upon the transition of emerin-null cells from day 0 to day 1 (cell-cycle withdrawal). The transition to myocyte commitment during emerin-null progenitor differentiation caused 709 transcripts to be differentially expressed in emerin-null progenitors (Figure 2B; day 1 to day 2). Few differentially expressed transcripts were seen during emerin-null myotube formation and maturation, as 32 transcripts and 7 transcripts were differentially expressed from day 2 to day 3 and day 3 to day 4, respectively (Figure 2B). There were an average of 29,337 ± 629 transcripts identified in each comparison. All differentially expressed transcripts had *q*-value <0.05.

**Figure 3:**
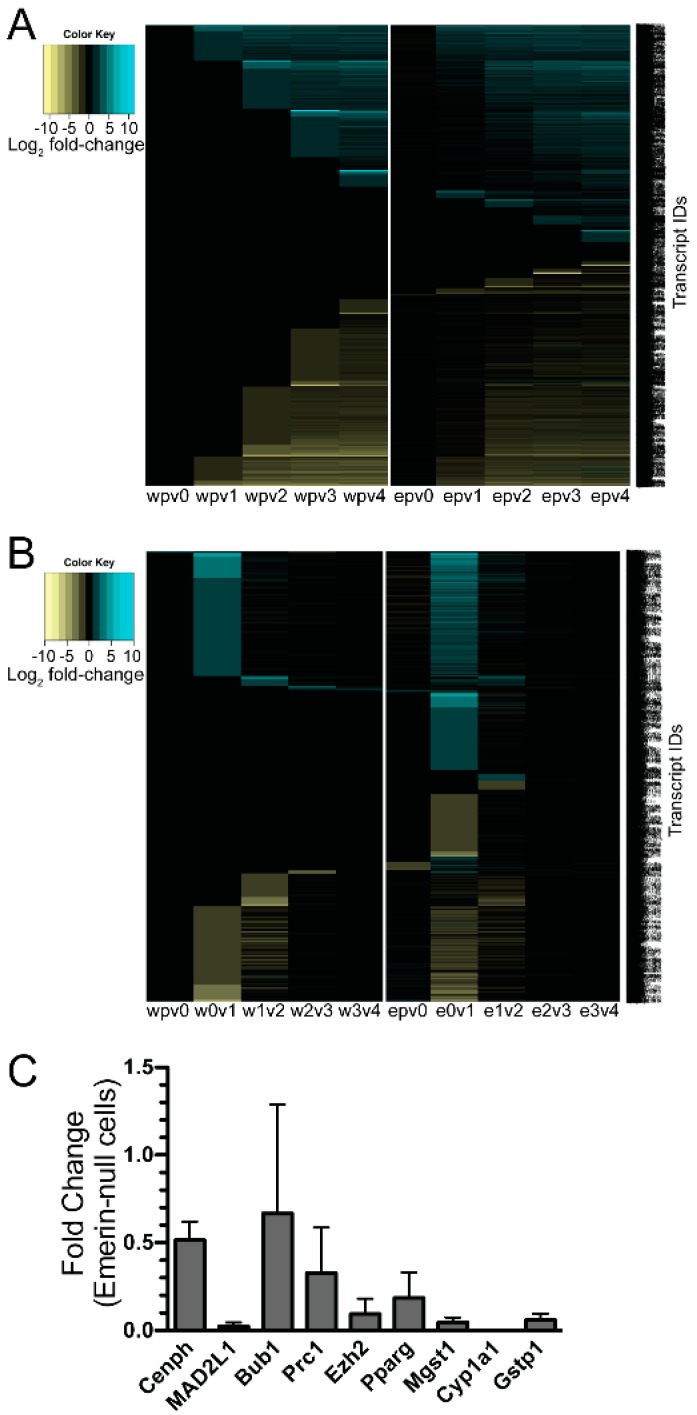
Thousands of genes were differentially expressed during differentiation of emerin-null myogenic progenitors. (**A**) Heat map comparing wildtype and emerin-null cells to one another at each differentiation time point. (**B**) Heat map comparing gene expression changes for each day with the preceding day during differentiation of wildtype (w) or emerin-null (e) myogenic progenitors. Expression changes are Log_2_. Yellow signifies downregulation; Blue signifies activation; e, emerin-null; w, wildtype; v, versus; p, proliferating cells, 0, differentiation day 0; 1, differentiation day 1; 2, differentiation day 2; 3, differentiation day 3; 4, differentiation day 4. The names of the >3000 transcripts are shown on the right. (**C**) qPCR validation of selected genes. RNA was purified from differentiating emerin-null or wildtype myogenic progenitors one day after differentiation induction. Fold change refers to changes in expression in emerin-null cells compared to wildtype cells. Expression of each gene in emerin-null cells was normalized to GAPDH and Oaz1. Data represents mean and SEM.

The goal of these studies was to determine how the coordinated temporal gene expression program was altered in emerin-null myogenic progenitors as compared to wildtype progenitors to cause impaired differentiation. To follow the differentially expressed transcript expression throughout differentiation the data was plotted as heat map (Figure 3A). Some of the transcripts differentially expressed in emerin-null cells compared to wildtype cells remain constant throughout differentiation. After one day of differentiation there is a significant compression in the number of differentially expressed transcripts (Figure 2A and Figure 3A), revealing a significant and unique complement of primarily upregulated transcripts (Figure 2A and Figure 3A) during emerin-null differentiation. This agrees with growing evidence that the nuclear envelope is a generally repressive domain, with the loss of an INM protein, such as emerin, expected to be associated with loss of repression for a subset of genes in the domain, and suggests that emerin-null cells accumulate expression differences from their wildtype counterparts as they move further into differentiation.

**Figure 4:**
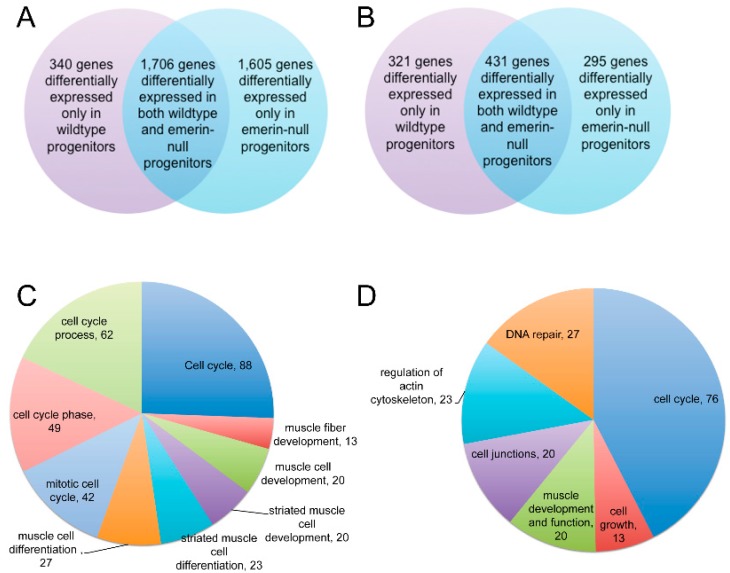
A large number of differentially expressed genes were present only in differentiating emerin-null or wildtype cells. (**A**) Venn diagram of differentially expressed genes during the transition from differentiation day 0 to day 1 showing transcripts differentially expressed in both wildtype and emerin-null cells or differentially expressed only in differentiating wildtype or emerin-null cells. (**B**) Venn diagram of differentially expressed genes during the transition from differentiation day 1 to day 2 showing transcripts differentially expressed in both wildtype and emerin-null cells or differentially expressed only in differentiating wildtype or emerin-null cells. (**C**) Pie Chart showing distribution of significant gene ontology (GO)-terms comparing wildtype and emerin-null cells in the transition from day 0 to day 1 of differentiation derived from DAVID analysis of differentially expressed genes unique to emerin-null cells. (**D**) Pie Chart showing distribution of significant GO-terms comparing wildtype and emerin-null cells in the transition from day 1 to day 2 of differentiation derived from DAVID analysis of differentially expressed genes unique to emerin-null cells.

We predicted a more powerful analysis would be the comparison of transcripts differentially expressed at important differentiation transitions during differentiation of emerin-null and wildtype progenitors. We anticipated this analysis would yield insight into the genes involved in the inability of emerin-null progenitors to properly differentiate. To identify transcripts that were differentially expressed only in differentiating wildtype or emerin-null progenitors at each transition point, the differentially expressed genes between each day and the preceding day were determined for either wildtype or emerin-null differentiating progenitors. The results were then plotted as a heat map (Figure 3B), in which changes in transcript expression could be easily seen throughout differentiation. Importantly, this also showed there were many transcripts that were aberrantly expressed at specific transitions during emerin-null myogenic differentiation. It was these gene expression differences that are likely important for the impaired differentiation of emerin-null myogenic progenitors. For example, transcripts that are differentially expressed during wildtype differentiation between day 0 and day 1 of differentiation are identified. Differentially expressed transcripts were then identified during the transition from differentiation day 0 to day 1 in emerin-null cells. These two datasets were then compared to each other to determine the genes that are uniquely differentially expressed in wildtype cells or emerin-null cells during the transition from day 0 to day 1 of differentiation (Figure 4A). This approach showed a large number of transcripts were differentially expressed between day 0 and day 2 of emerin-null and wildtype progenitor differentiation (Figure 2B and Figure 3B), as expected, since this is when the differentiation program is initiated. Most changes in transcript expression between wildtype and emerin-null progenitors occurred during the transition from day 0 to day 1 of differentiation (Figure 3B), which is when the myogenic progenitors exit the cell cycle. 3651 transcripts were differentially expressed during wildtype and emerin-null differentiation days 0 and 1, with 1706 transcripts altered in both wildtype and emerin-null progenitors (Figure 4A; *q*-value < 0.05). 1605 transcripts were differentially expressed only in emerin-null progenitors; 340 transcripts were differentially expressed in wildtype progenitors. This transition coincides with cell cycle exit, suggesting the failure of emerin-null progenitors is caused by failure in transcriptional reprogramming at this crucial transition state. 1047 transcripts showed altered expression during the transition from day 1 to day 2 of myogenic differentiation (Figure 4B; *q*-value < 0.05). 431 transcripts showed altered expression in both wildtype and emerin-null cells. 295 transcripts were differentially expressed only in emerin-null cells during this transition, whereas 321 transcripts were differentially expressed only in wildtype cells (*q*-value < 0.05).

Overall, these results show the robust nature of myogenic differentiation, with a large number of myogenic genes expressed early during differentiation to transcriptionally reprogram the cells in response to the strong myogenic cue of serum withdrawal and initiate myogenic differentiation. These changes persist until approximately day 2 (Figure 3B), suggesting the differentiation program is set at this point in these myogenic progenitors. This reprogramming appears to be dysfunctional in emerin-null myogenic progenitors, as a large number of genes are differentially expressed between emerin-null and wildtype progenitors from day 0 to day 2.

### 3.4. qPCR Was Done to Validate the Results from the RNAseq Analysis

Although validation is often unnecessary for RNAseq, as the RNAseq technology has been used and validated for many years, we still performed quantitative RT-PCR (qPCR) to validate selected transcripts. We randomly selected *Cenph*, *Mad2l1*, *Bub1*, *Prc1*, *Ezh2*, *Pparg*, *Mgst1*, *Cyp1a1* and *Gstp1* for validation. The transition from day 0 to day1 of differentiation in emerin-null myogenic progenitors was used for validation by qPCR. RNA sequencing of these samples showed *Cenph*, *Mad2l1*, *Bub1*, *Prc1*, *Ezh2*, *Pparg*, *Mgst1*, *Cyp1a1* and *Gstp1* were downregulated 5.10-fold, 1.91-fold, 1.65-fold, 1.71-fold, 1.80-fold, 10.0-fold, 3.08-fold, 16.6-fold and 1.92-fold, respectively. All of these changes in transcript expression were validated by qPCR, although the magnitude of downregulation sometimes varied by 2- to 5-fold between RNAseq and qPCR. *Cenph*, *Mad2l1*, *Bub1*, *Prc1*, *Ezh2*, *Pparg*, *Mgst1*, *Cyp1a1* and *Gstp1* were downregulated 1.95-fold, 43.5-fold, 1.5-fold, 3.1-fold, 10.6-fold, 5.45-fold, 21.7-fold, 870-fold and 16.7-fold, respectively (Figure 3C).

### 3.5. Pathway and Network Analysis of Differentially Expressed Transcripts during Myogenic Differentiation of Wildtype and Emerin-Null Progenitors

To identify the molecular networks and pathways that fail to be reprogrammed in emerin-null progenitors during differentiation, we utilized the DAVID and STRING software platforms for gene ontology (GO) analysis and de novo pathway generation, respectively. The transition from day 0 to day 1 was chosen for the analysis because this is the time when myogenic progenitors exit the cell cycle and our data suggest this is where a majority of the transcriptional reprogramming occurs during differentiation of wildtype myogenic progenitors. Further, this transcriptional reprogramming appears to fail in emerin-null myogenic progenitors. Thus identifying the pathways containing these transcripts will likely provide us with the key molecular networks and pathways important for the impaired myogenic differentiation and regeneration seen in emerin-null myogenic progenitors and in patients.

Comparing the differences in transcript expression between differentiating wildtype and emerin-null progenitors at the transition from day 0 to day 1 of differentiation using DAVID gene ontology (GO) analysis of differentially expressed genes unique to emerin-null cells revealed 88 genes associated with the cell cycle, 62 genes associated with cell cycle processes, 49 genes associated with the cell cycle phase, 42 genes associated with mitotic cell cycle, 27 genes associated with muscle cell differentiation, 23 genes associated with striated muscle cell differentiation, 20 genes associated with striated muscle cell development, 20 genes associated with muscle cell development and 13 genes associated with muscle fiber development (Figure 4C). Thus during the day 0 to day 1 transition, the key myogenic differentiation transcriptional reprogramming transition, the differentially expressed transcripts unique to emerin-null cells or wildtype cells are enriched in pathways regulating cell cycle withdrawal. DAVID GO analysis of differentially expressed genes unique to emerin-null cells during the transition from day 1 to day 2 of differentiation enriched biological processes for cell cycle regulation, cell growth, DNA repair, cell junctions, muscle development and function and the actin cytoskeleton (Figure 4D).

**Figure 5:**
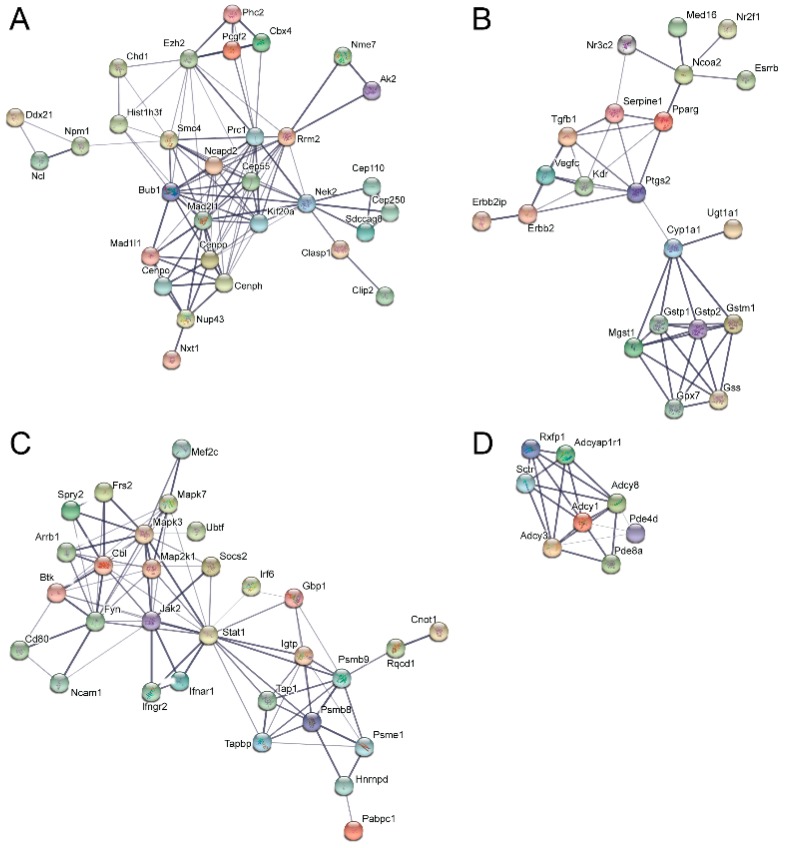
Identification of molecular interaction networks enriched for genes whose expression is altered during the transition from day 0 to day 1 in emerin-null myogenic differentiation. Differentially expressed genes present in only differentiating emerin-null progenitors during the transition from day 0 to day 1 in emerin-null cells were analyzed using STRING to identify interaction networks implicated in their impaired differentiation. The most enriched networks are shown and represent (**A**) cell cycle, (**B**) glutathione metabolism, (**C**) JAK-STAT signaling, and (**D**) G-protein and cAMP signaling.

**Figure 6:**
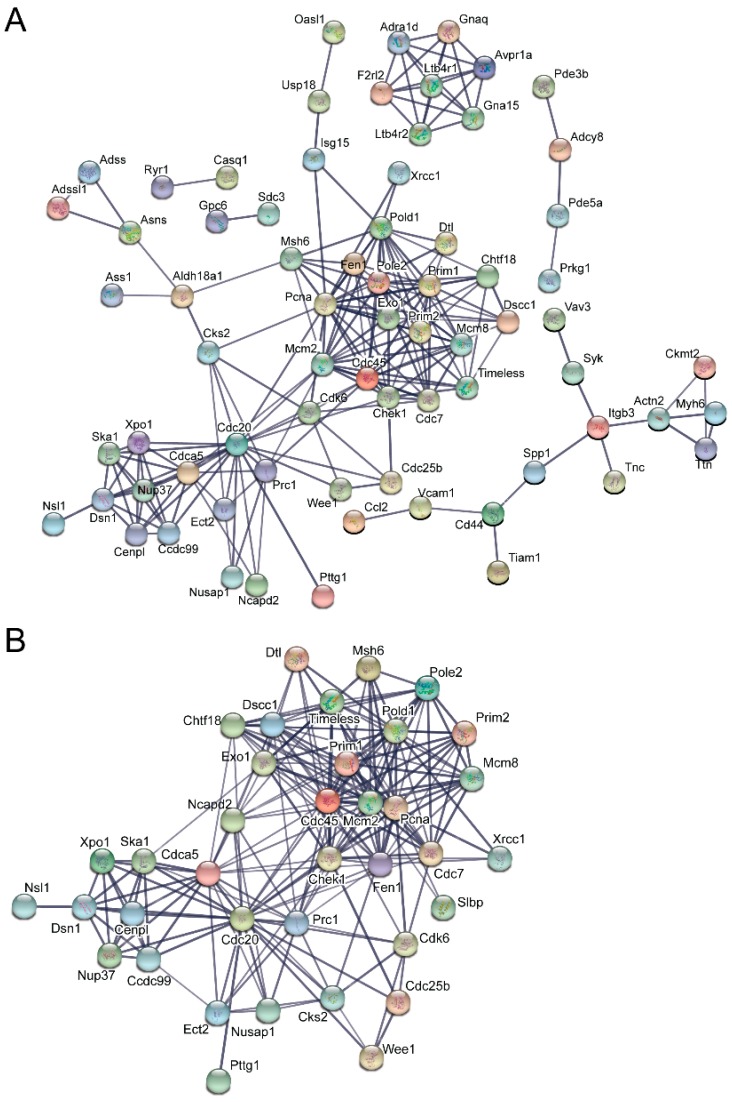
Identification of molecular interaction networks enriched for genes whose expression was altered during the transition from day 1 to day 2 in emerin-null myogenic differentiation. Differentially expressed genes present in only differentiating emerin-null progenitors during the transition from day 1 to day 2 in emerin-null cells were analyzed using STRING to identify interaction networks implicated in their impaired differentiation. (**A**) The most highly enriched and statistically significant and related interaction networks are shown. (**B**) The cell-cycle interaction network was the largest, most enriched network identified by STRING analysis on these samples.

STRING analysis, which is capable of generating interaction networks based on several different categories of protein-protein functional interaction data from their extensive database, found several interaction nodes from the differentially expressed transcripts found only in emerin-null cells during the day 0 to day 1 transition, including one containing numerous cell cycle genes (Figure 5A). Additionally, STRING analysis on this transition in emerin-null cells identified a node containing a number of cellular metabolism and redox-related proteins, suggesting that emerin-null cells may have difficulty responding to metabolic stress (Figure 5B). Multiple cell signaling networks were also enriched, including the JAK-STAT, G-protein and cAMP signaling pathways (Figure 5C,D). STRING analysis on differentially expressed transcripts only found in differentiating emerin-null myogenic progenitors during the transition from day 1 to day 2 identified a network enriched in cell cycle genes (Figure 6A,B), with smaller networks representing cell junctions, cAMP signaling, G-protein signaling and actin cytoskeleton (Figure 6A). STRING output does not indicate whether the expression of the genes indicated by the nodes are increased or decreased, but identifies interaction networks enriched for these genes and thus implicated in the impaired differentiation of emerin-null progenitors.

Ingenuity Pathway analysis (IPA) was also used to ensure a comprehensive analysis of the pathways and networks implicated in the impaired differentiation of emerin-null myogenic progenitors. This software is based on computer algorithms that analyze the functional connectivity of the genes from information obtained within the IPA database. The “core analysis” function feature in the software was used to interpret the differentially expressed data, which included biological processes, canonical pathways, upstream transcriptional regulators, and gene networks. Each gene identifier was mapped to its corresponding gene object in the Ingenuity Pathway Knowledge Base.

**Figure 7:**
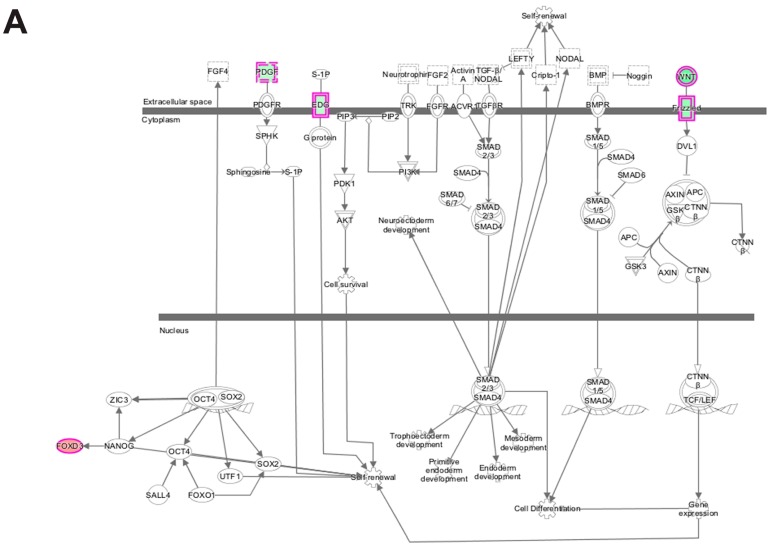

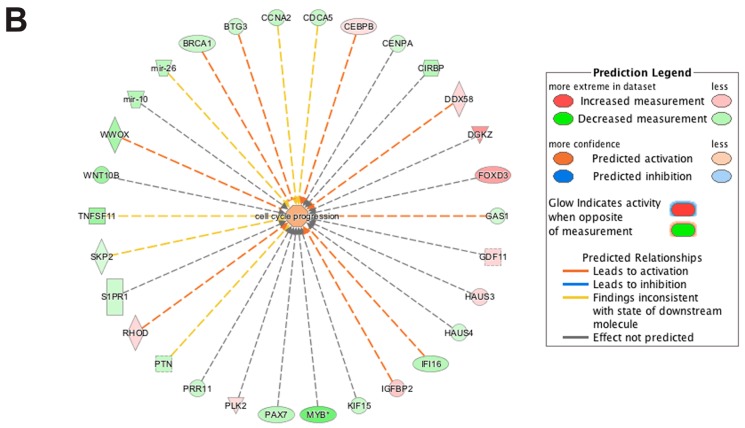
Ingenuity pathway analysis of differentially expressed genes unique to wildtype cells during transition from day 0 to day 1 of differentiation. (**A**) Human Embryonic Stem Cell Pluripotency was identified as a top canonical pathway containing differentially expressed genes unique to wildtype cells. The network was generated through the use of ingenuity pathway analysis (IPA) (Ingenuity Systems, www.ingenuity.com) on normalized mRNA values. Nodes represent molecules in a pathway, while the biological relationship between nodes is represented by a line (edge). Edges are supported by at least one reference in the Ingenuity Knowledge Base. The intensity of color in a node indicates the degree of up- (red) or down- (green) regulation. Nodes are displayed using shapes that represent the functional class of a gene product (Circle = Other, Nested Circle = Group or Complex, Rhombus = Peptidase, Square = Cytokine, Triangle = Kinase, Vertical ellipse = Transmembrane receptor). Edges are marked with symbols to represent the relationship between nodes (Line only = Binding only, Flat line = inhibits, Solid arrow = Acts on, Solid arrow with flat line = inhibits and acts on, Open circle = leads to, Open arrow = translocates to). (**B**) Biological Functional Analysis using IPA predicts activation of cell cycle progression from differentially expressed genes unique to wildtype cells during transition from day 0 to day 1 of differentiation (*p*-value: <0.05). The figure represents genes that are associated with a particular biological function that are altered in the uploaded dataset. Genes that are up-regulated are displayed within red nodes and those down-regulated are displayed within green nodes. The intensity of the color in a node indicates the degree of up-(red) or down-(green) regulation. The shapes of the nodes reflect the functional class of each gene product: transcriptional regulator (horizontal ellipse), transmembrane receptor (vertical ellipse), enzyme (vertical rhombus), cytokine/growth factor (square), kinase (inverted triangle) and complex/group/other (circle). An orange line indicates predicted upregulation, whereas a blue line indicates predicted downregulation. A yellow line indicates expression being contradictory to the prediction. Gray line indicates that direction of change is not predicted. Solid or broken edges indicate direct or indirect relationship, respectively.

Analysis of transcripts that are uniquely altered in differentiating wildtype progenitors during the transition from day 0 to day 1 identified Cdc42 signaling as a major canonical pathway in these cells along with Wnt/β-catenin signaling, calcium signaling, OX40 signaling pathway and Human Embryonic Stem Cell Pluripotency pathway (Figure 7A; Table 2). The Cdc42 signaling controls crucial cellular functions, which include cell morphology, migration, endocytosis and cell cycle progression (G1 to S phase) [44,45]. The human embryonic stem cell pluripotency pathway maintains embryonic cell in the proliferative and undifferentiated state. Oct4, SOX2 and Nanog are the major transcription factors that regulate pluripotency [46,47]. Wnt signaling also promotes pluripotency and Wnt signaling has well-established roles in maintaining satellite cell quiescence, satellite cell activation and myogenic differentiation after injury [26,32,33]. Wnt ligand and frizzled receptors were found to be down-regulated suggesting that the wildtype cells decrease their pluripotency potential through the Wnt pathway as they prepare to commit themselves to form differentiated myoblasts.

Functional enrichment analysis using IPA of transcripts uniquely altered in wildtype progenitors during the transition from day 0 to day 1 of differentiation predicted the activation of cell cycle progression and cell viability (Figure 7B). Other significantly enriched biological functions were G2/M phase transition, G2 phase, G1 phase (inhibition) and S phase (inhibition), all of which regulate specific phases of the cell cycle.

**Figure 8:**
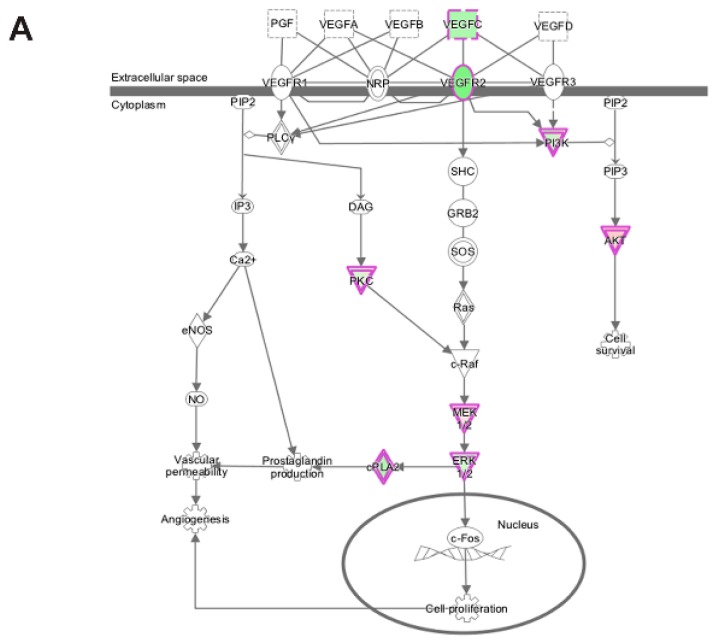

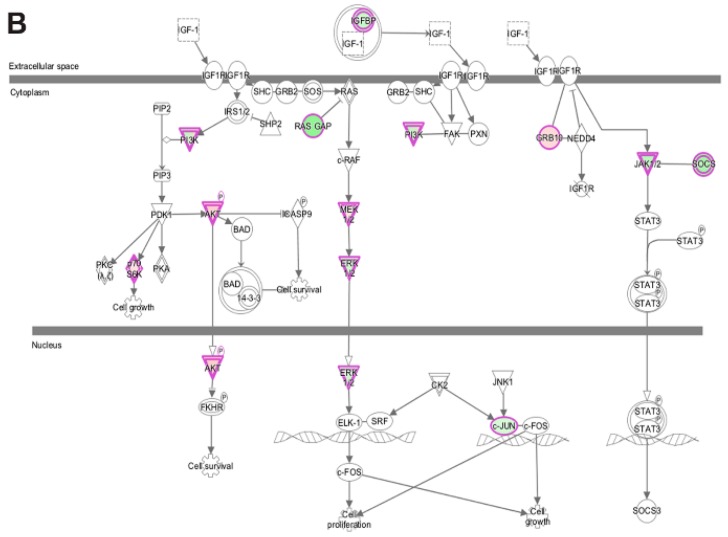
Ingenuity pathway analysis of differentially expressed genes unique to emerin-null cells during transition from day 0 to day 1 of differentiation. (**A**,**B**) IPA identified the VEGF (**A**) and IGF-1 (**B**) signaling pathways as enriched. The network was generated through the use of IPA (Ingenuity Systems, www.ingenuity.com) on normalized mRNA values. Nodes represent molecules in a pathway, while the biological relationship between nodes is represented by a line (edge). Edges are supported by at least one reference in the Ingenuity Knowledge Base. The intensity of color in a node indicates the degree of up- (red) or down- (green) regulation. Nodes are displayed using shapes that represent the functional class of a gene product (Circle = Other, Nested Circle = Group or Complex, Rhombus = Peptidase, Square = Cytokine, Triangle = Kinase, Vertical ellipse = Transmembrane receptor). Edges are marked with symbols to represent the relationship between nodes (Line only = Binding only, Flat line = inhibits, Solid arrow = Acts on, Solid arrow with flat line = inhibits and acts on, Open circle = leads to, Open arrow = translocates to).

Analysis of transcripts uniquely altered in differentiating emerin-null cells during the transition from day 0 to day 1 using the canonical pathways in IPA function found several pathways associated with cell proliferation, growth, signaling and metabolism were significantly enriched. Growth hormone signaling pathway (GH), STAT3 pathway, the HIPPO pathway, TGF-β signaling, IGF-1 signaling and VEGF signaling pathways were significantly inhibited during the transition from day 0 to day 1 in emerin-null cells (Table 2; Figure 8A,B). The VEGF and IGF signaling pathways (Figure 8A,B) are important for muscle differentiation and regeneration [29,30,31,35,48,49], as stimulation of the VEGF pathway increases myotube number, myogenic marker expression and myotube size in differentiated C2C12 cells [48]. IGF-1 signaling promotes satellite cell proliferation early in differentiation and promotes terminal differentiation of myocytes during skeletal muscle regeneration, suggesting loss of emerin causes quiescent satellite cells to be pushed towards an activated state, leading to depletion of the quiescent satellite cell niche [13]. The STAT3 pathway is known to regulate many other genes involved in cell cycle progression specifically during G1 to S phase transition [50]. Growth Hormone increases amino acid uptake into skeletal muscles, suggesting that this tissue is a primary target of the physiological effects of GH [51]. TGF-β is well documented to inhibit skeletal muscle satellite cell differentiation by inhibiting satellite cell proliferation, myofiber fusion and expression of key muscle-specific genes [25,52,53,54].

**Figure 9:**
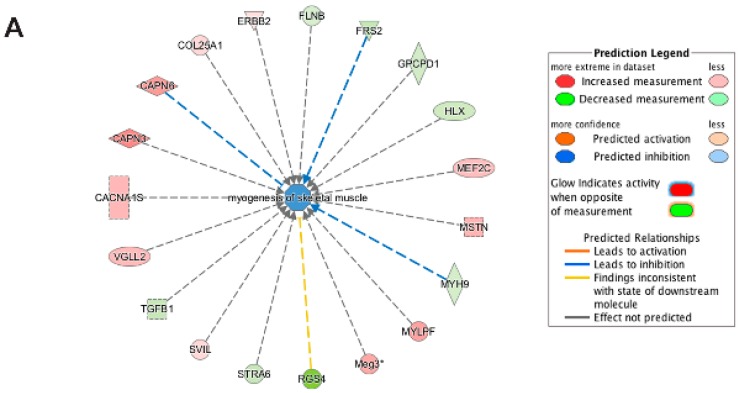

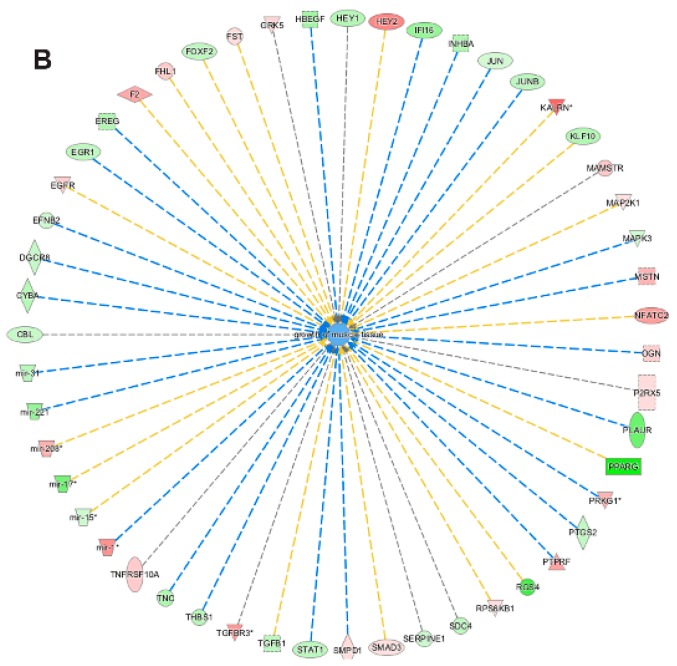
Biological Functional Analysis using Ingenuity pathway analysis of differentially expressed genes unique to emerin-null cells during transition from day 0 to day 1 of differentiation. (**A**,**B**) Biological Functional Analysis using IPA predicts inhibition of myogenesis of skeletal muscle from genes ((**A**) *p*-value: <0.001; *Z*-score: −1.067) and growth of skeletal muscle genes ((**B**) *p*-value: <0.001; *Z*-score: −0.799) as being highly enriched in the differentially expressed transcripts unique to emerin-null cells during transition from day 0 to day 1 of differentiation. The figure represents genes that are associated with a particular biological function that are altered in the uploaded dataset. Genes that are up-regulated are displayed within red nodes and those down-regulated are displayed within green nodes. The intensity of the color in a node indicates the degree of up-(red) or down-(green) regulation. The shapes of the nodes reflect the functional class of each gene product: transcriptional regulator (horizontal ellipse), transmembrane receptor (vertical ellipse), enzyme (vertical rhombus), cytokine/growth factor (square), kinase (inverted triangle) and complex/group/other (circle). An orange line indicates predicted upregulation, whereas a blue line indicates predicted downregulation. A yellow line indicates expression being contradictory to the prediction. Gray line indicates that direction of change is not predicted. Solid or broken edges indicate direct or indirect relationship, respectively.

Functional enrichment analysis of transcripts differentially expressed only in emerin-null progenitors during the transition from day 0 to day 1 of differentiation were enriched for biological functions associated with myogenesis of skeletal muscles and growth of muscle tissue (Figure 9A,B), as well as proliferation of muscle cells and formation of muscle and myogenesis (not shown); all of these functions were predicted to be inhibited during day 0 to day 1 of differentiation. This unbiased analysis of emerin-null differentiation identifies putative pathways involved in the delayed commitment to differentiation due to prolonged retention in the cell cycle after 24 h of differentiation induction seen in these cells [11].

**Figure 10:**
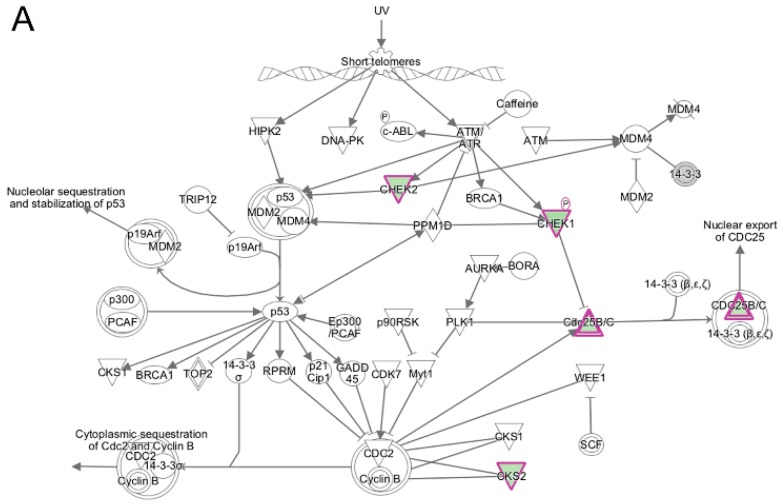

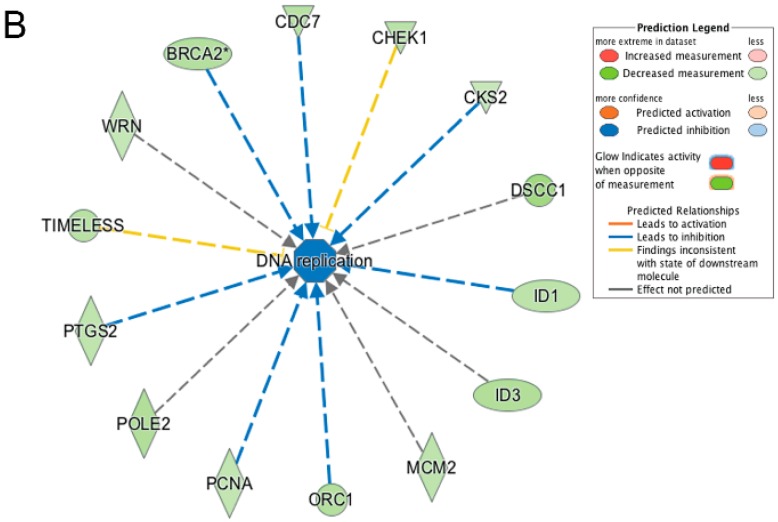
Ingenuity pathway analysis of differentially expressed genes unique to wildtype cells during transition from day 1 to day 2 of differentiation. (**A**) The top canonical pathway enriched from genes differentially expressed only in wildtype cells was the Cell Cycle: G2/M DNA Damage Checkpoint Regulation pathway. The network was generated through the use of IPA (Ingenuity Systems, www.ingenuity.com) on normalized mRNA values. Nodes represent molecules in a pathway, while the biological relationship between nodes is represented by a line (edge). Edges are supported by at least one reference in the Ingenuity Knowledge Base. The intensity of color in a node indicates the degree of up- (red) or down- (green) regulation. Nodes are displayed using shapes that represent the functional class of a gene product (Circle = Other, Nested Circle = Group or Complex, Rhombus = Peptidase, Square = Cytokine, Triangle = Kinase, Vertical ellipse = Transmembrane receptor). Edges are marked with symbols to represent the relationship between nodes (Line only = Binding only, Flat line = inhibits, Solid arrow = Acts on, Solid arrow with flat line = inhibits and acts on, Open circle = leads to, Open arrow = translocates to). (**B**) Biological Function Analysis with IPA on genes differentially expressed only in wildtype cells during transition from day 1 to day 2 of differentiation identified DNA replication (*p*-value: <0.05) as the top pathway. The figure represents genes that are associated with a particular biological function that are altered in the uploaded dataset. Genes that are upregulated are displayed within red nodes and those down-regulated are displayed within green nodes. The intensity of the color in a node indicates the degree of up-(red) or down-(green) regulation. The shapes of the nodes reflect the functional class of each gene product: transcriptional regulator (horizontal ellipse), transmembrane receptor (vertical ellipse), enzyme (vertical rhombus), cytokine/growth factor (square), kinase (inverted triangle) and complex/group/other (circle). An orange line indicates predicted upregulation, whereas a blue line indicates predicted downregulation. A yellow line indicates expression being contradictory to the prediction. Gray line indicates that direction of change is not predicted. Solid or broken edges indicate direct or indirect relationship, respectively.

Similar to what was seen in the DAVID analysis, when IPA was used to analyze the transcripts unique to differentiating wildtype progenitors during the transition from day 1 to day 2, cell cycle pathways and kinase signaling were the top canonical pathways identified (Table 3). These included G2/M DNA damage checkpoint regulation (Figure 10A), cell cycle control of chromosomal replication, mitotic roles of Polo-Like Kinase and ILK signaling. Polo-like kinases were reported to be involved in regulating mitosis at various key steps [55,56], including mitotic exit and the DNA damage checkpoint. Functional enrichment analysis in IPA of these same transcripts showed enrichment for biological functions associated with DNA replication (Figure 10B), M-phase and S-phase and cell cycle progression (not shown); all of these functions were predicted to be inhibited during day 1 to day 2 differentiation. The enrichment of cell cycle regulatory pathways clearly show that cellular mechanisms regulating cell cycle withdrawal are likely functioning only in the differentiating wildtype myogenic progenitors during this transition, since these pathway components were found only in the wildtype cells. This further supports our hypothesis that the failure of differentiating emerin-null myogenic progenitors to activate or repress these pathways during differentiation significantly contributes to their impaired differentiation.

**Figure 11:**
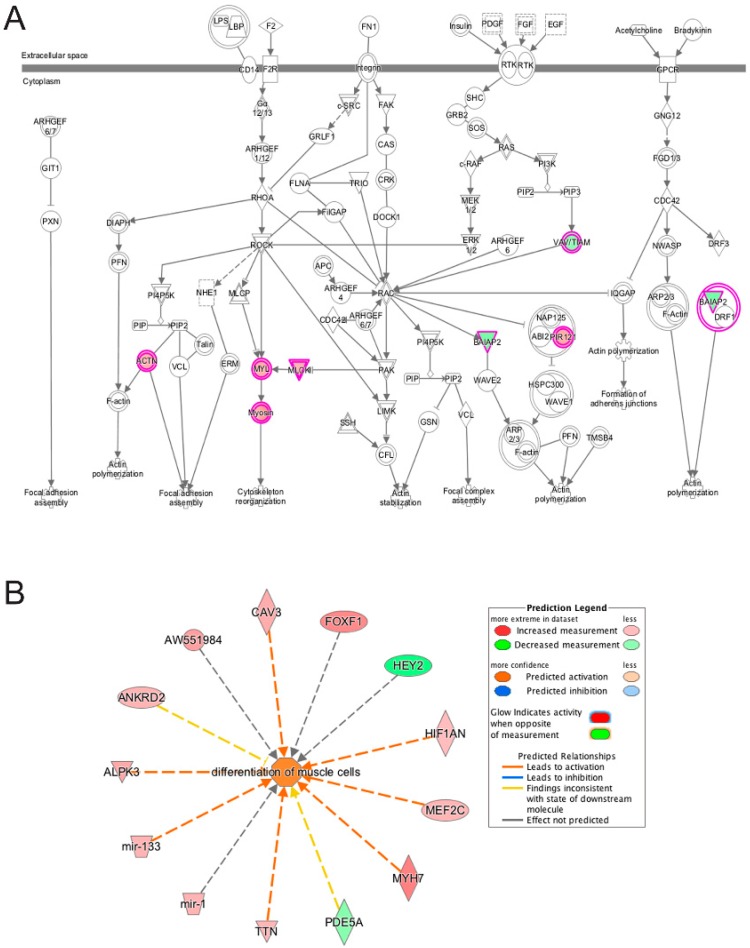
Ingenuity pathway analysis of differentially expressed genes unique to emerin-null cells during transition from day 1 to day 2 of differentiation. A, The Actin-Cytoskeleton signaling pathway was identified as a top canonical pathway in emerin-null cells. The network was generated through the use of IPA (Ingenuity Systems www.ingenuity.com) on normalized mRNA values. Nodes represent molecules in a pathway, while the biological relationship between nodes is represented by a line (edge). Edges are supported by at least one reference in the Ingenuity Knowledge Base. The intensity of color in a node indicates the degree of up- (red) or down- (green) regulation. Nodes are displayed using shapes that represent the functional class of a gene product (Circle = Other, Nested Circle = Group or Complex, Rhombus = Peptidase, Square = Cytokine, Triangle = Kinase, Vertical ellipse = Transmembrane receptor). Edges are marked with symbols to represent the relationship between nodes (Line only = Binding only, Flat line = inhibits, Solid arrow = Acts on, Solid arrow with flat line = inhibits and acts on, Open circle = leads to, Open arrow = translocates to). B, Biological Function Analysis with IPA on genes differentially expressed only in emerin-null cells during the transition from day 1 to day 2 of differentiation identified the activation of differentiation of muscle cells (*p*-value: <0.001; *Z*-score: 1.263). The figure represents genes that are associated with a particular biological function that are altered in the uploaded dataset. Genes that are up-regulated are displayed within red nodes and those down-regulated are displayed within green nodes. The intensity of the color in a node indicates the degree of up-(red) or down-(green) regulation. The shapes of the nodes reflect the functional class of each gene product: transcriptional regulator (horizontal ellipse), transmembrane receptor (vertical ellipse), enzyme (vertical rhombus), cytokine/growth factor (square), kinase (inverted triangle) and complex/group/other (circle). An orange line indicates predicted upregulation, whereas a blue line indicates predicted downregulation. A yellow line indicates expression being contradictory to the prediction. Gray line indicates that direction of change is not predicted. Solid or broken edges indicate direct or indirect relationship, respectively.

Netrin signaling, Calcium signaling, integrin signaling and axonal guidance signaling pathways were enriched in differentially expressed transcripts unique to emerin-null cells during the transition from day 1 to day 2 (Table 2). Actin cytoskeleton signaling is involved in most, if not all of these pathways, and was also highly enriched by IPA (Figure 11A). Netrin signaling was previously shown to be important for myogenic differentiation and myofiber size both in vitro and in vivo by regulating phosphorylation of ERK [57,58,59]. Integrin signaling was previously shown to be important during myogenic differentiation and skeletal muscle function, as mice lacking α7β1, the predominant integrin complex during differentiation, have impaired regeneration and exhibit a dystrophic phenotype [60,61]. Integrins were also shown to be important for myoblast fusion and terminal differentiation [62]. Calcium signaling and calcium homeostasis have long been known to be important for skeletal muscle function and regeneration [63]. Functional enrichment analysis of these transcripts were enriched for biological functions associated with differentiation of muscle cells (Figure 11B), as well as organization of cytoplasm, contractility of muscle, muscle contraction, organization of actin cytoskeleton, microtubule dynamics, cellular homeostasis, abnormal morphology of muscle and myogenesis of skeletal muscle (not shown). Further, actin cytoskeleton signaling, actin cytoskeletal rearrangements, and integrin and adherens junction signaling is known to be important for muscle regeneration in vivo when the myoblasts must adhere to the myofiber, rearrange its cytoskeleton and fuse with the myofiber.

**Figure 12:**
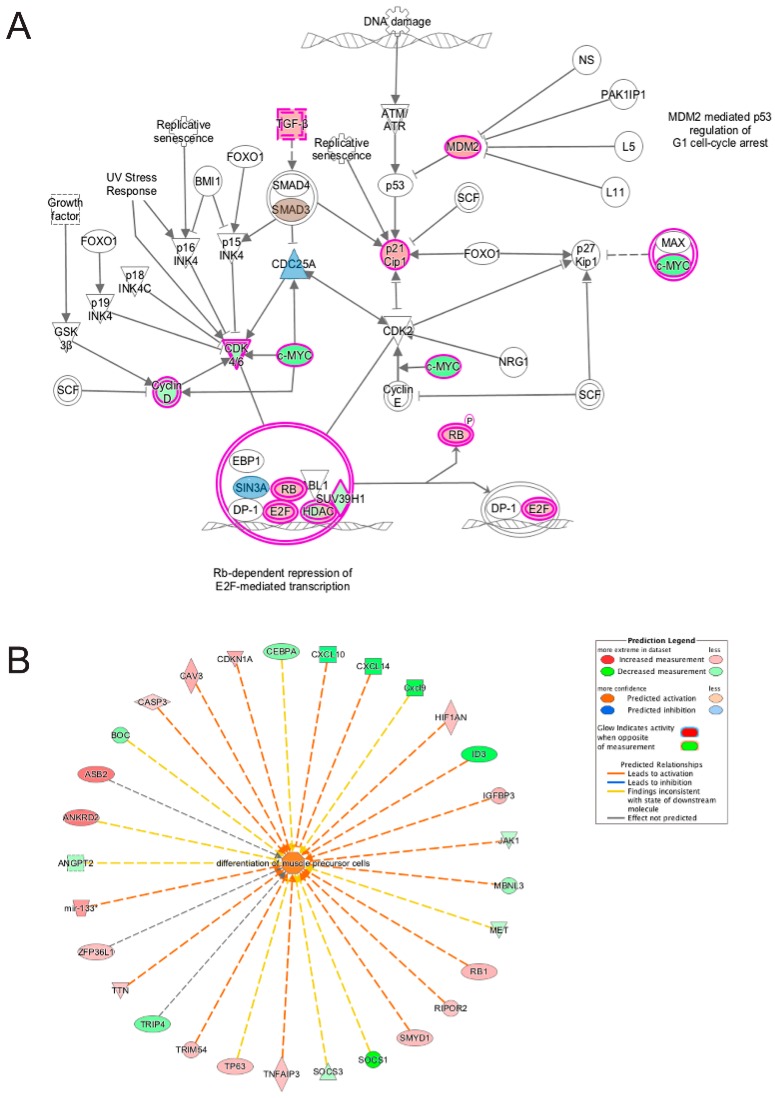
Ingenuity pathway analysis of genes differentially expressed during the transition from differentiation day 0 to day 1 common to emerin-null cells and wildtype cells. (**A**) The Cell Cycle G1/S checkpoint regulation pathway is significantly enriched in both wildtype and emerin-null cell during transition from day 0 to day 1 of differentiation. The network was generated through the use of IPA (Ingenuity Systems, www.ingenuity.com) on normalized mRNA values. Nodes represent molecules in a pathway, while the biological relationship between nodes is represented by a line (edge). Edges are supported by at least one reference in the Ingenuity Knowledge Base. The intensity of color in a node indicates the degree of up- (red) or down- (green) regulation. Brown colored nodes (up-regulation) and blue colored nodes (down-regulation) indicate additional nodes in the pathway that are uniquely altered in emerin-null cells. Nodes are displayed using shapes that represent the functional class of a gene product (Circle = Other, Nested Circle = Group or Complex, Rhombus = Peptidase, Square = Cytokine, Triangle = Kinase, Vertical ellipse = Transmembrane receptor). Edges are marked with symbols to represent the relationship between nodes (Line only = Binding only, Flat line = inhibits, Solid arrow = Acts on, Solid arrow with flat line = inhibits and acts on, Open circle = leads to, Open arrow = translocates to). (**B**) Biological Function Analysis predicts activation of differentiation of muscle precursor cells in both wildtype cells and emerin-null cells during transition from day 0 to day 1 of differentiation (*p*-value: <0.001; *Z*-score: 1.350). The figure represents genes that are associated with a particular biological function that are altered in the uploaded dataset. Genes that are up-regulated are displayed within red nodes and those down-regulated are displayed within green nodes. The intensity of the color in a node indicates the degree of up-(red) or down-(green) regulation. The shapes of the nodes reflect the functional class of each gene product: transcriptional regulator (horizontal ellipse), transmembrane receptor (vertical ellipse), enzyme (vertical rhombus), cytokine/growth factor (square), kinase (inverted triangle) and complex/group/other (circle). An orange line indicates predicted upregulation, whereas a blue line indicates predicted downregulation. A yellow line indicates expression being contradictory to the prediction. Gray line indicates that direction of change is not predicted. Solid or broken edges indicate direct or indirect relationship, respectively.

IPA was also used to analyze differentially expressed transcripts that were present in both emerin-null and wildtype myogenic progenitors during the transition from day 0 to day 1 of differentiation. Transcripts that were altered in opposite directions were noted, although this was a minor number of genes. Therefore, the magnitude of each differentially expressed gene shared between emerin-null and wildtype cells at the transition from day 0 to day 1 was analyzed to identify components whose expression was significantly different between wildtype and emerin-null cells. These components were then mapped to canonical pathways to identify pathways implicated in the impaired differentiation of emerin-null progenitors. The G1/S cell cycle checkpoint pathway was highly enriched (Table 3; Figure 12A). This was not surprising given the failure of emerin-null cells to properly exit the cell cycle upon differentiation induction. Although expression of the transcripts represented in these pathways were all in the same direction, we identified additional nodes in this pathway that were represented by differentially expressed transcripts that were unique to emerin-null cells at this transition. These included downregulation of TGF-β1 by 2-fold, SMAD7 by 3-fold, Cdc25a by 1.8 fold and Sin3a by 1.8 fold, and activation of SMAD3 by 1.7 fold (Figure 12A). These changes are predicted to affect Rb repression of E2F-mediated transcription and withdrawal from the cell cycle specifically in differentiating emerin-null cells. Functional pathways enriched in these transcripts were unsurprisingly those involved in differentiation of muscle progenitors, myogenic differentiation and muscle development (Figure 12B).

**Figure 13:**
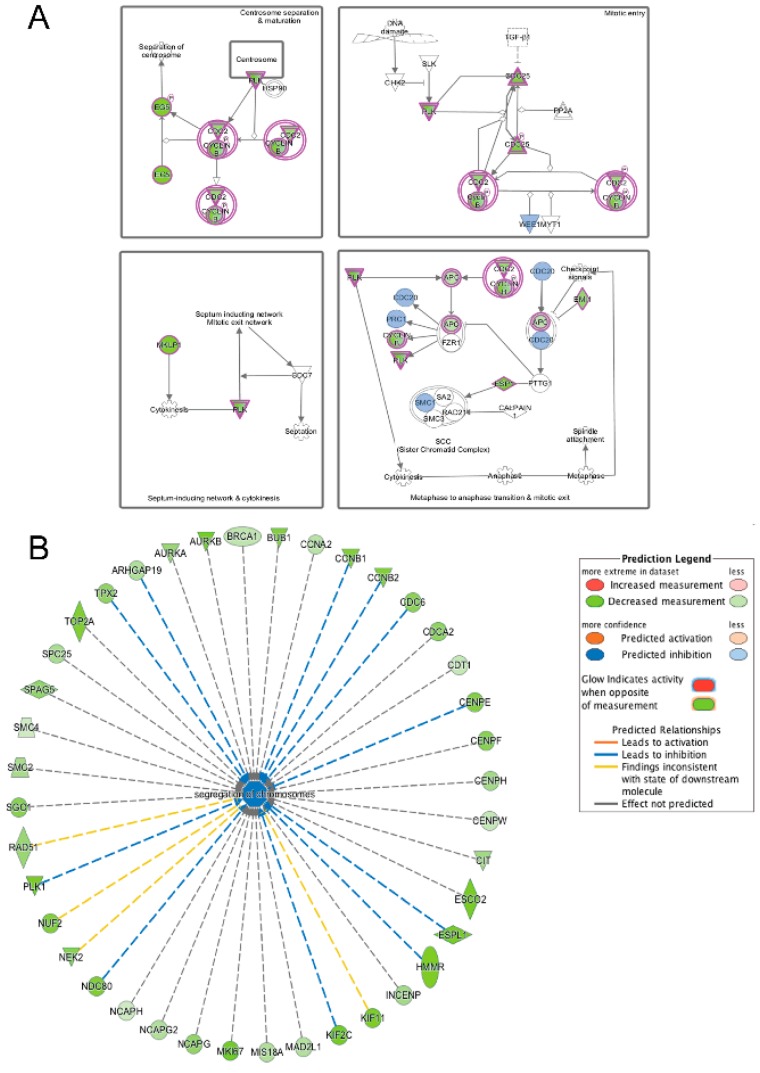
Ingenuity pathway analysis of genes differentially expressed during the transition from differentiation day 1 to day 2 common to emerin-null cells and wildtype cells. (**A**) Mitotic roles of polo-like kinase pathway is significantly enriched in both wildtype and emerin-null cells during the transition from day 1 to day 2 of differentiation. The network was generated through the use of IPA (Ingenuity Systems, www.ingenuity.com) on normalized mRNA values. Nodes represent molecules in a pathway, while the biological relationship between nodes is represented by a line (edge). Edges are supported by at least one reference in the Ingenuity Knowledge Base. The intensity of color in a node indicates the degree of up- (red) or down- (green) regulation. Brown colored nodes (up-regulation) and blue colored nodes (down-regulation) indicate additional nodes in the pathway that are uniquely altered in emerin-null cells. Nodes are displayed using shapes that represent the functional class of a gene product (Circle = Other, Nested Circle = Group or Complex, Rhombus = Peptidase, Square = Cytokine, Triangle = Kinase, Vertical ellipse = Transmembrane receptor). Edges are marked with symbols to represent the relationship between nodes (Line only = Binding only, Flat line = inhibits, Solid arrow = Acts on, Solid arrow with flat line = inhibits and acts on, Open circle = leads to, Open arrow = translocates to). (**B**) Biological Function Analysis enriched inhibition of segregation of chromosomes pathway among those genes commonly differentially expressed among wildtype cells and emerin-null cells during transition from day 1 to day 2 of differentiation (*p*-value: <0.001; *Z*-score: −2.000). The figure represents genes that are associated with a particular biological function that are altered in the uploaded dataset. Genes that are up-regulated are displayed within red nodes and those down-regulated are displayed within green nodes. The intensity of the color in a node indicates the degree of up-(red) or down-(green) regulation. The shapes of the nodes reflect the functional class of each gene product: transcriptional regulator (horizontal ellipse), transmembrane receptor (vertical ellipse), enzyme (vertical rhombus), cytokine/growth factor (square), kinase (inverted triangle) and complex/group/other (circle). An orange line indicates predicted upregulation, whereas a blue line indicates predicted downregulation. A yellow line indicates expression being contradictory to the prediction. Gray line indicates that direction of change is not predicted. Solid or broken edges indicate direct or indirect relationship, respectively.

IPA was also used to analyze differentially expressed transcripts that were present in both emerin-null and wildtype myogenic progenitors during the transition from day 1 to day 2 of differentiation. Cell cycle pathways, mitosis and checkpoint pathways were highly enriched. Although expression of all of the transcripts represented in these pathways was in the same direction, the magnitude of their expression was often very different. Emerin-null cells during the transition from day 1 to day 2 of differentiation had 5.3-fold less Eg5, 6.5-fold less PLK, 3.2-fold less cyclin B, 6.5-fold less Esp1, and 3-fold less Cdc25 (Figure 13A). Additional nodes in this pathway that are uniquely altered in wildtype cells or emerin-null cells were also identified. Transcripts unique to wildtype cells included Chk2, Pttg1, and Cdc7, which were downregulated 3-fold, 8.6-fold and 4-fold, respectively. Transcripts unique to emerin-null cells included Wee1, Cdc20, Prc1 and Smc1a which were downregulated 3-fold, 4.9-fold, 4.9-fold and 1.9 fold, respectively. Functional pathways enriched in shared transcripts from the transition from day 1 to day 2 of differentiation were cell cycle pathways (Figure 13B). Collectively, the changes in the direction and magnitude of transcript expression in differentiating emerin-null progenitors are predicted to result in a failure of the progenitors to exit the cell cycle and instead to enter mitosis.

## 4. Discussion

We previously found perturbations in the expression of components of the Notch, Wnt, IGF and TGF-β signaling pathways by monitoring gene expression using microarray technology in proliferating myogenic progenitor cells. To confirm these perturbations in proliferating progenitors using a newer, less biased technology, and to test if these pathways were disrupted throughout differentiation, we performed high-throughput RNA sequencing in myogenic cells during differentiation. The results of these experiments were partially in agreement with our work in proliferative cells, including changes to canonical myogenic signaling pathways. Additionally, our experiments revealed extensive divergence in transcript expression for emerin-null and wildtype cells during day 0 to day 2 of myogenic differentiation.

The number of transcripts differentially expressed between wildtype and emerin-null cells at each day of myogenic differentiation varies significantly. Differential expression between the two cell lines decreases substantially at day 1 of myogenic differentiation, likely due to the emerin-null cells ‘catching-up’ and attempting to initiate transcriptional reprograming at the onset of myogenic differentiation, as seen in wildtype cell. It is clear that this transcriptional reprogramming is incomplete, since at each subsequent transition during differentiation, a large number of different genes become differentially expressed, with a marked trend towards overexpression of transcripts in emerin-null cells, consistent with a less repressive nuclear environment. This suggests that expression differences between wildtype and emerin-null cells do not remain consistent throughout myogenic differentiation. Our previous work in proliferating wildtype and emerin-null myogenic progenitor cells showed disruptions to the Notch, Wnt, IGF and TGF-β signaling pathways [13]. Emerin-null cells continued to show disruptions in expression of genes involved in these canonical myogenic signaling pathways, as IGF-related, TGF-β related genes and Wnt-related genes were differentially expressed throughout myogenic differentiation in emerin-null cells. Disruptions to Notch signaling were less apparent; however, due to the delicate relationship between these major myogenic signaling pathways, further investigation into perturbations to these canonical myogenic signaling pathways may be important for understanding the progression of pathology in emerin-null cells.

Our comparisons of gene expression during myogenic differentiation transition points within wildtype or emerin-null cells revealed that the most extensive changes to transcription during myogenic differentiation appear to take place within the day 0 to day 1 transition, with a lesser but significant number of changes occurring during the day 1 to day 2 transition. Many of these changes are shared between wildtype and emerin-null cells, however a significant number of differentially expressed transcripts in the day 0 to day 1 transition were unique to either cell type. These genes are likely to contain a number of promising candidate genes and pathways for understanding EDMD disease etiology. With many myogenic genes correctly expressed during differentiation, especially in response to the strong myogenic cue of serum withdrawal, this demonstrates the extremely robust nature of myogenic differentiation.

Analyzing this data further, a key interval during myogenic differentiation was identified as the transition from day 0 to day 1 of differentiation, during which the largest number of different transcriptional changes between the two lines occur. We predict this transition demarcates a transcriptional reprograming event required to activate myogenic differentiation. These analyses also revealed the cumulative nature of expression differences between wildtype and emerin-null cells; after the onset of myogenic differentiation, there is a contraction in expression differences between wildtype and emerin-null cells at day 1 of myogenic differentiation, with steady increases in differential transcript expression thereafter. A large number of differentially expressed transcripts were seen from the transition from day 1 to day 2, when committed myogenic progenitors (myoblasts) get cues to begin aligning. This suggested that the emerin-null progenitors may be playing catch-up and are expressing genes that are normally present in the transition from day 0 to day 1. Alternatively, these differentially expressed transcripts may illustrate the failure of differentiating emerin-null progenitors to align properly, which is necessary for myotube formation. The decrease in differentially expressed genes in days 2–4 suggests that either the differentiating emerin-null progenitors have caught up or the majority of transcriptional reprograming ultimately failed, but myotube formation still occurred. Transcriptional reprogramming occurring during cell cycle exit leads to activation of the differentiation program and repression of the proliferative program. This reprogramming fails in emerin-null progenitors leading to failure in cell cycle exit and impaired differentiation. This defective transcriptional reprogramming is predicted to be caused by the failure of emerin-null progenitors to coordinate the temporal reorganization of their genome properly during differentiation. This would explain the large number of transcripts differentially expressed in differentiating emerin-null progenitors.

This work revealed molecular pathways implicated in the impaired differentiation of emerin-null progenitors, both those previously suggested by other studies and those that have not been, previously reported. Previous studies showed impaired differentiation of emerin-null myoblasts may have resulted from altered Rb signaling leading to delayed cell cycle exit [6,8,11,13]. Our results confirm this finding, but fail to show any alterations in the Rb pathway. However, we do find many other cell cycle pathways altered, including those containing key components of cell cycle checkpoints, such as cdc25, wee1 and cyclin B. Others and we also showed the ERK and p38 pathways were important for differentiation of emerin-null myogenic progenitors [10,11,36,64]. The results presented here confirm these findings. During myogenesis in emerin-null cells, we found altered expression of genes involved in cellular metabolism, including those involved in glutathione metabolism. Recent evidence showed the expression of genes involved in glutathione metabolism was also altered in flies expressing EDMD-causing mutants [65] and represented alterations in the ability of the cells to respond to oxidative stress. Our findings yield new insight into the extent to which loss of emerin affects cell signaling, as many additional, previously unreported pathways were significantly enriched by this analysis, including VEGF signaling, embryonic stem cell signaling, G2M checkpoint signaling, integrin signaling, actin-mediated cytoskeletal signaling, HIPPO pathway, DNA damage, mitotic pathways, Ox40 signaling, Cdc42 signaling, and many more pathways. Many of the nodes are shared amongst these pathways and thus it will be important to interrogate these pathways individually using pharmacological activators and inhibitors. Importantly, these findings suggest novel avenues through which pharmacological agents, such as cell cycle inhibitors or redox agents, may be helpful in restoring wildtype function to emerin-null cells. Further work will be needed to establish how emerin regulates these molecular pathways to determine the best molecular targets for therapeutic intervention. Additionally, this work has generated a powerful resource for further analysis of transcription during differentiation in primary wildtype and emerin-null myogenic cells.

The studies presented here used a cell-based system to follow differentiation, in which myotubes are formed by myoblast-to-myoblast fusion or myoblast-to-myotube fusion. In vivo, skeletal muscle regeneration is more complex. Only under severe muscle damage will myoblast-myoblast fusion occur, as myogenic progenitors will attach to the extracellular matrix remnants and myoblasts will fuse to one another or to the newly forming myotubes [66,67]. Under mild trauma, the satellite (myogenic progenitor) cells will become activated and begin differentiating, move to the site of the injury and begin fusing to the intact fiber to repair the damage.

The repair mechanisms for mild damage to skeletal muscle use similar pathways to those used upon sever damage, including the Notch, IGF-1, TGF-β, VEGF, Platelet Derived Growth Factor (PDGF) and Fibroblast Growth Factor (FGF) pathways [67]. In the initial phase of repair to mild damage, there is necrosis and inflammation, remodeling of the ECM and removal of the debris, new fiber formation, remodeling of the vasculature and innervation. The IGF-1 and TGF-β pathways play key roles in the remodeling phase, as well as in satellite cell activation, myoblast proliferation and commitment to differentiation [68,69,70]. Macrophages act in this initial phase to remove debris and secrete pro-inflammatory molecules that also cause myoblast proliferation (Lu et al., 2011), followed by release of anti-inflammatory molecules that stimulate myoblast differentiation and fusion [71,72,73]. Vascularization and angiogenesis is stimulated by VEGF release by differentiating myoblasts [71,74,75]. Conversely, release of VEGF, IGF-1 and FGF from endothelial cells stimulates the growth of satellite cells and proliferation of myogenic progenitors [74].

Importantly, many of the molecular pathways used during skeletal muscle repair in vivo were altered during emerin-null myogenic progenitor differentiation. IGF-1, TGF-β, VEGF, PDGF and FGF pathways were all significantly enriched by this analysis and were predicted to be attenuated. This suggests emerin-null progenitors would likely fail to respond to endothelial cell stimulation of progenitor proliferation and differentiation. Attenuated IGF-1 signaling would also be predicted to impair skeletal muscle regeneration in vivo by failing to activate myogenic progenitors and failing to promote terminal differentiation of myocytes [13]. The JAK-STAT pathway was also significantly enriched as uniquely differentially expressed in differentiating emerin-null progenitors. JAK-STAT signaling is important for satellite cell expansion and its aberrant activation reduces satellite cell numbers and impairs myogenic differentiation [76,77,78]. Differentiating emerin-null progenitors were enriched for genes in multiple cell cycle pathways and they failed to withdraw from the cell cycle upon differentiation induction. Withdrawal from the cell cycle is required for proliferating myogenic progenitors to commit to differentiation and proceed with the differentiation program both in vitro and in vivo.

### How Does Loss of Emerin Cause Such Massive Changes in Gene Expression?

We predict the failure of emerin-null cells to properly reorganize the genome upon receipt of transcriptional reprogramming signals on serum withdrawal results in these large changes. There is mounting evidence supporting emerin function in genomic organization at the nuclear periphery. This includes the organization of gene loci containing important differentiation genes (Pax3/7, MyoD, Myf5) whose expression must be temporally coordinated for myogenic differentiation to occur normally. Based upon our previous work, we predict the binding of emerin to HDAC3 and the subsequent activation of HDAC3 catalytic activity [12,21] is one of the primary mechanisms underlying recruitment and maintenance of repressed genomic regions to the nuclear lamina and their subsequent reorganization upon transcriptional reprogramming. It is possible emerin acts merely as a molecular tether after HDAC3-mediated repression of target loci. Due to the activation of HDAC3 by emerin, we consider this unlikely. We predict the improper epigenetic regulation of developmental loci by HDAC3 precedes the signaling defects and differentiation delay seen in emerin-null cells. Theophylline was shown to directly stimulate HDAC3 activity in emerin-null progenitors and rescue the temporal localization and expression of Pax3/7, MyoD, and Myf5. Recently theophylline was recently shown to partially rescue emerin-null progenitor differentiation [11]. In this way, epigenetic regulation was able to be uncoupled from emerin’s other functions to support the hypothesis that increasing HDAC3 activity rescues the coordinated gene repression required during differentiation. It will be important to test whether theophylline rescues transcriptional reprograming upon differentiation induction to exhibit a transcriptional profile resembling differentiating wildtype cells. As emerin also binds to and modulates the activity of multiple transcriptional regulators [18,20,23,79,80], the possibility that disruption of these functional interactions may also contribute to their impaired differentiation.

Other nuclear envelope proteins may play similar roles or analogous roles at later time points during differentiation. Similar results were seen for the inner nuclear envelope protein nuclear envelope transmembrane protein 39 (NET39) during C2C12 differentiation. NET39 becomes activated upon differentiation induction and is maximally expressed at days 1 and 2 of C2C12 differentiation [81,82,83]. Similar to emerin-null cells, decreased NET39 expression impairs differentiation by altering the genomic organization of genomic loci containing myogenic differentiation genes, including genes to be activated (e.g., muscle genes) and genes to be repressed (e.g., cell cycle genes) [83]. Thus we hypothesize emerin and NET39, as well as potentially other INM proteins, cooperate to coordinate the temporal genomic reorganization during differentiation to coordinate the transcriptional reprogramming necessary for commitment to differentiation. Further we predict emerin acts during the initial receipt of the transcriptional reprograming signal and the myogenic commitment transition, while we predict NET39 acts to ‘maintain’ the differentiated state by coordinating the transitions of committed myoblasts to fully differentiated myotubes.

Collectively, these data suggest alteration of the signaling pathways and biological functions identified in the emerin-null cells may be responsible for the impaired differentiation and regeneration seen in EDMD patients. This could also help explain the relatively late onset of skeletal muscle wasting seen in EDMD as progressive loss of the satellite cell niche is predicted to cause a relatively slow loss of muscle regeneration over time. Further studies using pharmacological inhibitors or activators of these important signaling pathways would be examined to test if normal signaling pathway functions can be restored in emerin-null myogenic progenitors. Using this approach, we predict potential drugs will be identified to treat EDMD patients.

## Figures and Tables

**Table 1:** List of qRT-PCR primers used in this study.

Gene	Forward (5’-3’)	Reverse (5’-3’)
Cenph	ACATACATTCCAGGGCCTTATT	CTGCAGAGGATGCCACTTTA
Mad2l1	AAGTCCGTCTACGCTCATTTAC	CTCAGACAAGTCCAGGAAGAAC
Bub1	TGGGTTCTTTGCTGGTCTATG	CCCTACTAATATGCTGCCATTCT
Prc1	CCTCTTCTGGTGTGCAGAAATA	CAAGAAACCCTCACTGGGATAG
Ezh2	CAGCTCAAGAGGTTCAGAAGAG	GGGCGACCAAGAGTACATTATAG
Pparg	CTGGCCTCCCTGATGAATAAAG	AGGCTCCATAAAGTCACCAAAG
Mgst1	ACCGCATTCCAGAGGATAAC	CGTCAGTGCGAACAAACTTC
Cyp1a1	GTGAGCAAGGAGGCTAACTATC	GGCTACTGACACGACCAAATA
Gstp1	GAGACCTCACCCTTTACCAATC	CCCATCATTCACCATATCCATCT
Gapdh	AACATTGGCATTGTGGAAGGGCTC	TGGAAGAGTGGGAGTTGCTGTTGA
Oaz1	GAGCTGAATGCTGTGTTTGTC	AGGTCACCTGACCATCTTAAAC

**Table 2:** List of Enriched Canonical Pathways from genes unique to either wildtype cells or emerin-null cells during key differentiation transitions using Ingenuity Pathway Analysis.

Canonical Pathways Identified Unique to Wildtype Progenitors during Transition from Day 0 to Day 1 of Differentiation	Canonical Pathways Identified Unique to Emerin-Null Progenitors during Transition from Day 0 to Day 1 of Differentiation	Canonical Pathways Identified Unique to Wildtype Progenitors during Transition from Day 1 to Day 2 of Differentiation	Canonical Pathways Identified Unique to Emerin-Null Progenitors during Transition from Day 1 to Day 2 of Differentiation
OX40 Signaling Pathway	Growth Hormone Signaling	Cell cycle control of chromosomal replication	Netrin signaling
(*p*-value: <0.001)	(*p*-value: <0.001)	(*p*-value: <0.05)	(*p*-value: <0.001)
Cdc42 Signaling	STAT3 pathway	Cell cycle: G2/M DNA Damage Checkpoint Regulation	Calcium Signaling
(*p*-value: <0.001)	(*p*-value: <0.01)	(*p*-value: <0.05)	(*p*-value: <0.001)
Epoxysqualene Biosynthesis	TGF β Signaling	Actin Cytoskeleton Signaling	Actin cytoskeleton signaling
(*p*-value: <0.001)	(*p*-value: <0.01)	(*p*-value: <0.05)	(*p*-value: <0.001)
Glycine Biosynthesis I	EGF Signaling	Mitotic Roles of Polo-Like Kinase	Mitotic Roles of Polo-Like Kinase
(*p*-value: <0.05)	(*p*-value: <0.01)	(*p*-value: <0.05)	(*p*-value: <0.001)
Human Embryonic Stem Cell Pluripotency	HIPPO Pathway	ILK signaling	Axonal guidance signaling
(*p*-value: <0.05)	(*p*-value: <0.05)	(*p*-value: <0.05)	(*p*-value: <0.01)
Transcriptional Regulatory in Embryonic Stem cells	Glutamate Signaling	TGF β Signaling	Integrin signaling
(*p*-value: <0.05)	(*p*-value: <0.05)	(*p*-value: <0.05)	(*p*-value: <0.05)
	IGF-1 Signaling		
	(*p*-value: <0.05)		
	VEGF Signaling		
	(*p*-value: <0.05)		

**Table 3:** List of Enriched Canonical Pathways from genes commonly shared among wildtype cells and emerin-null cells during key differentiation transitions using Ingenuity Pathway Analysis.

Canonical Pathway Identified for Genes Common between Wildtype and Emerin-Null Cells during Transition from Day 0 to Day 1 of Differentiation	Canonical Pathway Identified for Genes Common between Wildtype and Emerin-Null Cells during Transition from Day 1 to Day 2 of Differentiation
cell cycle control of chromosomal replication	cell cycle control of chromosomal replication
(*p*-value: <0.001)	(*p*-value: <0.001)
ILK signaling	ATM signaling
(*p*-value: <0.001)	(*p*-value: <0.001)
Integrin signaling	Mitotic roles of polo-like kinase
(*p*-value: <0.01)	(*p*-value: <0.001)
Cell Cycle G1/S checkpoint regulation	Cell Cycle G2/M DNA damage checkpoint regulation
(*p*-value: <0.01)	(*p*-value: <0.001)
P38 MAPK signaling	Estrogen mediated S phase entry
(*p*-value: <0.01)	(*p*-value: <0.001)
Cdc42 Signaling	Role of CHK Proteins in Cell Cycle checkpoint control
(*p*-value: <0.01)	(*p*-value: <0.001)
Mitotic roles of polo-like kinase	Cyclins and cell cycle Regulation
(*p*-value: <0.01)	(*p*-value: <0.001)
p53 signaling	p53 signaling
(*p*-value: <0.05)	(*p*-value: <0.001)
PAK signaling	Aryl Hydrocarbon Receptor Signaling
(*p*-value: <0.05)	(*p*-value: <0.001)
